# Larval Dispersal of *Spodoptera frugiperda* Strains on Bt Cotton: A Model for Understanding Resistance Evolution and Consequences for its Management

**DOI:** 10.1038/s41598-017-16094-x

**Published:** 2017-11-23

**Authors:** José B. Malaquias, Wesley A. C. Godoy, Adriano G. Garcia, Francisco de S. Ramalho, Celso Omoto

**Affiliations:** 1 Department of Entomology and Acarology, Luiz de Queiroz College of Agriculture (ESALQ), University of São Paulo (USP), Av. Pádua Dias 11, Piracicaba, 13418-900 São Paulo, Brazil; 2 Embrapa Algodão, Oswaldo Cruz, 1.143, Centenário, 58428-095 Campina Grande-PB, Brazil

## Abstract

High dispersal of Lepidoptera larvae between non-Bt and Bt cotton plants can favour the evolution of insect resistance; however, information on host acceptance of neonates in tropical transgenic crops is scarce. Therefore, the purposes of this study were as follows: (*i*) to investigate the feeding behaviour of susceptible and Cry1F-resistant strains of *Spodoptera frugiperda* (J.E. Smith) on Bt and non-Bt cotton (*Gossypium hirsutum* L.) varieties and (*ii*) to understand the possible effects of cotton field contamination on the dispersal and infestation capacity of *S*. *frugiperda* larvae by using an individual-based model. The main results of this paper are as follows: (*1*) the highest post-feeding larval dispersal of the Cry1F-resistant strain occurred at an exposure time of 18–24 h; (*2*) via video tracking assays, we found that the least distance moved was by larvae resistant to Cry1F on non-Bt cotton; and (*3*) the model indicated differences in mobility capacity between Bt and non-Bt cotton. We conclude that resistant neonates exhibit sedentary behaviour. Our report represents the first findings concerning the fitness cost of larval behaviour traits of *S*. *frugiperda* associated with Cry1F resistance in Brazilian populations.

## Introduction

The cropping system in Brazil is intensive, which favours the overlapping of generations of insect pests such as *Spodoptera frugiperda* (J. E. Smith) (Lepidoptera: Noctuidae). This overlap increases the selection for resistance to insecticides and transgenic crops expressing *Bacillus thuringiensis* Berliner (Bt) toxins due to gene flow between *S*. *frugiperda* populations from maize and cotton fields in the same geographical region^1^. Another challenge to resistance management is seed contamination. Contamination may occur for many reasons, especially if Bt seeds, non-Bt seeds or both are contaminated by mixing seeds or by gene flow between cotton varieties via pollen flow^2^. In developing countries such as Brazil, the presence of volunteer plants, cross contamination between different plant species, or gene flow between Bt and non-Bt varieties, coupled with the practice of harvesting and saving seeds for use in the following year, are common causes for concern. Depending on the contamination degree and whether larvae move among plants within a field, resistance evolution may be accelerated^3^. In addition, insect-resistant genetically modified crops can affect some behavioural traits, including locomotion and feeding behaviour, as well as larval dispersal^4^.

The resistance of *S*. *frugiperda* to insecticides and Bt toxins has been characterized in Brazil under laboratory and field conditions^5–8^. Therefore, there is an urgent need to implement resistance management strategies. In recent decades, the demand for systematic information about the behavioural ecology of *S*. *frugiperda* on transgenic crops to explain the reasons for control failure has increased. With potential seed contamination, the movement from non-Bt plants to Bt plants within a field may promote insect survival in the last instars and consequently favour resistance evolution^9^; in this condition, the main reason for larval survival on Bt plants in the last instars is the brief exposure time during which the insect does not ingest a sufficient amount of insecticidal proteins to cause mortality before metamorphosis to the pupal stage^10^. This behaviour was observed in *S*. *frugiperda* larvae that were fed non-Bt cotton for 18 days and were subsequently transferred to Bt cotton expressing Cry1Ac and Cry1F toxins, where they reached pupal and adult stages^9^. Similarly, *S*. *frugiperda* strains in the last instars survived on Bt maize expressing Vip3Aa20 and Cry1Ab^10^.

The incorporation of knowledge about the movement of insect pests within large areas is essential for pest management, especially due to the lack of information on where, when, and why these pests move^11^. Larval dispersal has been investigated considering risk formulation of seed mixing because when larvae are stimulated by Bt plants, they may move a longer distance than expected towards non-Bt cotton plants, and resistance could evolve within a few years^12^. Knowledge about the movement of Lepidoptera larvae between Bt and non-Bt cotton plants is based on the configuration of the refuge^13,14^. Pre-feeding dispersal and post-feeding dispersal (*PFD*) of neonates is common when the host contains toxins, as observed from studies with Bt^15^. For pests with low mobility capacity, the seed mixture may provide a refuge comparable to a structured refuge, leading to longer delays to resistance^13^. Nevertheless, for pests with a high dispersal capacity, further studies to determine the consequences of the feeding behaviour of pests on Bt crops are needed, especially under conditions of non-structured or different degrees of seed contamination.

Theoretical models that consider the movement of Lepidoptera larvae are relevant for predicting the population dynamics of pests in agricultural systems, allowing one to study the effects of different refuge proportions and the movements of larvae on cotton crops with various resistance allele frequencies^16^. Modelling is a necessary procedure to guide professionals in designing effective programmes for resistance management in different scenarios and a helpful tool to explain the efficacy of a technology according to the landscape structure, i.e., the refuge, and to evaluate the potential consequences of contamination of Bt and non-Bt cotton fields.

Computational models that integrate information about the ecological traits of pest strains are necessary, especially in tropical conditions. The inclusion of information about ecological traits in modelling is essential to explain the fast resistance evolution of *S*. *frugiperda* to Bt cotton in Brazilian agroecosystems or similar systems. Although the fitness costs were not evaluated for the biological parameters of Cry1F-resistant strains of *S*. *frugiperda* ^17^ collected from Brazilian agroecosystems, the formulation of a hypothesis based on the possible existence of differences in feeding behaviour between larval phenotypes motivates the comparison of these ecological traits between susceptible and Cry1F-resistant strains of *S*. *frugiperda* on Bt cotton. Therefore, this study had two main questions of interest. Does the feeding behaviour differ between susceptible and Cry1F-resistant strains of *S*. *frugiperda*? Does the fitness cost associated with Cry1F resistance in *S*. *frugiperda* affect the spatial pattern in accordance with contamination of Bt and non-Bt cotton? In the context of the importance of assessing the feeding behaviour and neonate dispersal of *S*. *frugiperda* on Bt and non-Bt cotton, this study aimed to quantify some behavioural ecological traits of *S*. *frugiperda* larvae on Bt cotton and to investigate the effects of larval movement on population distribution. The overall goal was to gain an understanding of the rapid evolution of resistance and the possible implications for resistance management based on these behavioural traits. We studied the following variables: dispersal rate, *PFD*, individual proportions that were found on varieties and feeding on plant tissues (*IFP*), survival (via a host acceptance bioassay), distance moved, mean velocity, and continuous mobility period (via a video tracking assay). Larval dispersal and larval density on Bt and non-Bt cotton were also spatially simulated using an individual-based model.

## Results

### Feeding behaviour of *S*. *frugiperda* strains on WideStrike and non-Bt cotton varieties

We selected two components of the eigenvalues of the correlation matrix according to the criterion of Kaiser^18^. The first principal component (PC1) is represented by the contrast between the variables dispersal rate (eigenvector = 0.7026 *x*_1_) and *IFP* (eigenvector = 0.5577 *x*_2_). PC2 includes the contrast among *PFD* (0.4469 *z*_1_), the weighted average of *IFP* (eigenvector = −0.5958 *z*_2_) and survival (eigenvector = −0.6429 *z*_3_). PC1 and PC2 explain 39.42 and 33.79% of the total variation in the data, respectively.

**Figure 1:**
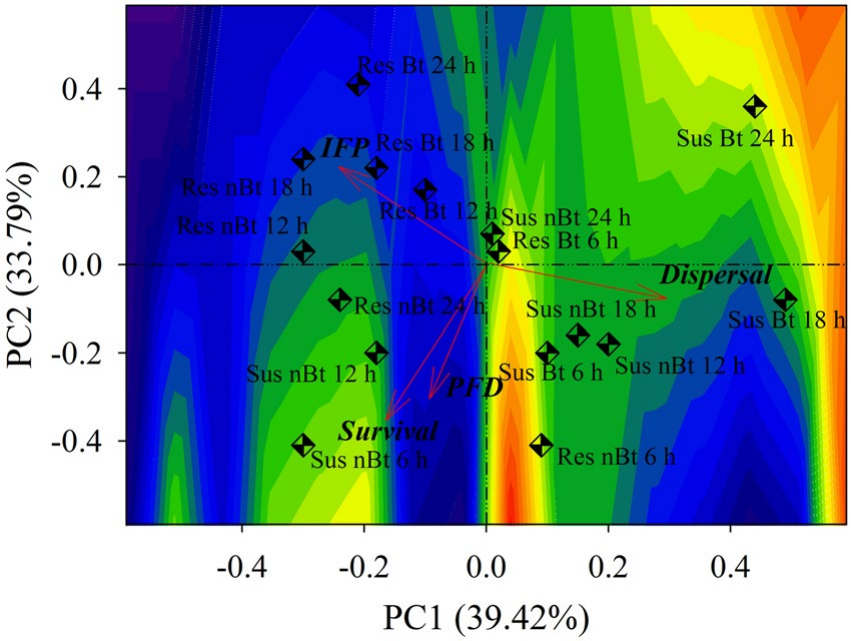
Biplot of variables: survival, dispersal rate, post-feeding dispersal (*PFD*) and individual proportions of *Spodoptera frugiperda* neonate that were found on varieties and feeding on plant tissues (*IFP*). The dots represent the following treatments: larvae of susceptible strain on Bt cotton (Sus Bt) and non-Bt cotton (Sus nBt) and Cry1F-resistant larvae on Bt cotton (Res Bt) and non-Bt cotton (Res nBt) at the time intervals 0–6 h, 6–12 h, 12–18 h, and 18–24 h. Contour area was defined based on the eigenvectors of each component.

The results of Pearson’s correlation analysis and the arrangement of the vectors in the biplot showed a negative correlation (Pearson’s correlation coefficient = −0.5253) between dispersal and *IFP*. Characterizing treatments via biplot enables us to determine the highest survival rate and greatest *PFD* for susceptible insects that were kept on non-Bt cotton plants for 6 and 12 h and for Cry1F-resistant insects that were kept on non-Bt cotton plants for 6 h. A tendency towards a low dispersal rate and a high proportion of plant tissue feeding was revealed on the biplot for the Cry1F-resistant insects at 12, 18, and 24 h of exposure to Bt cotton and at 12 and 18 h of exposure to non-Bt cotton (Fig. 1).

**Figure 2:**
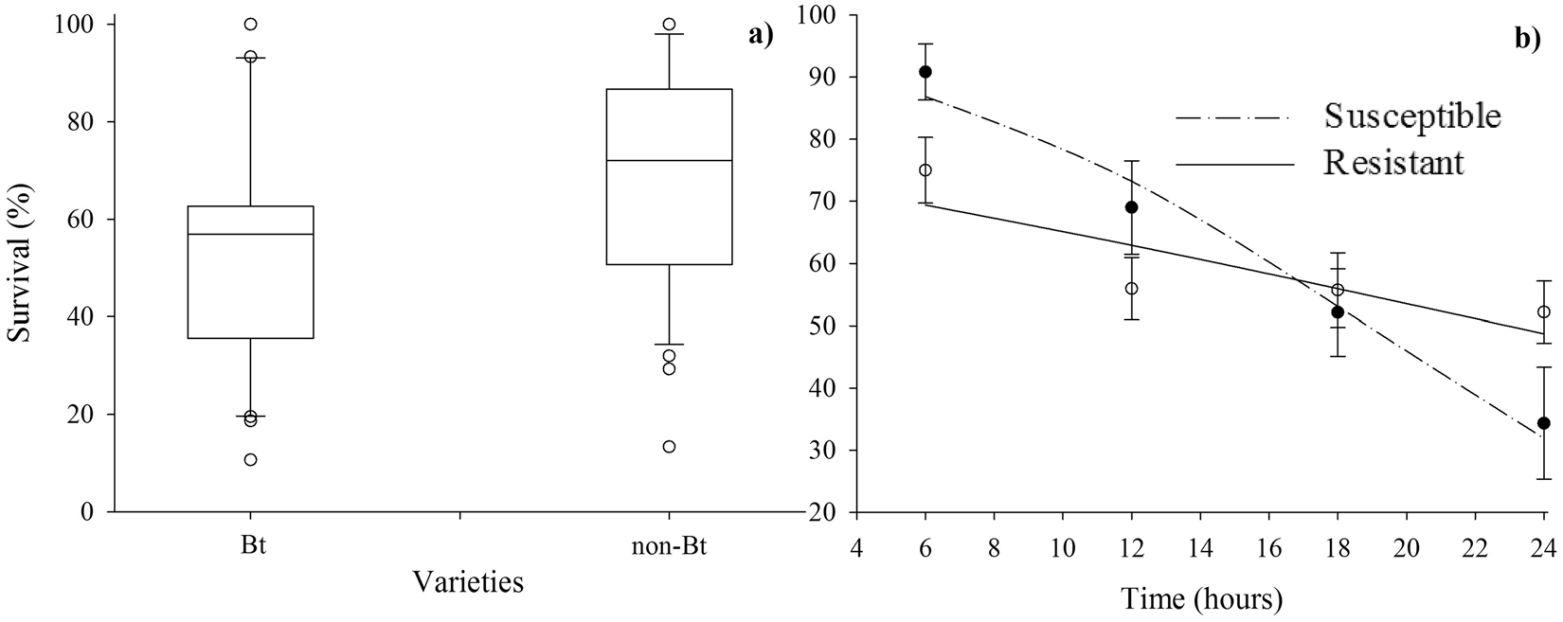
(**a**) Boxplot of *Spodoptera frugiperda* survival (%) on Bt (WideStrike) and non-Bt cotton. (**b**) Survival (%) (mean ± SE) of susceptible (exp-0.1470time + 2.7690/1 + exp-0.1470time + 2.7690) and Cry1F-resistant (exp-0.0483time + 1.1089/1 + exp-0.0483time + 1.1089) strains as a function of time. Original data.

Larval survival differed significantly between cotton varieties (*F*_1,46_ = 11.90; *P* = 0.0410) (Table 1 and Fig. 2a). The boxplot in Fig. 2a shows the effect of cotton varieties on the survival of *S*. *frugiperda*. Larval survival was greater than 70% on non-Bt cotton and lower than 60% on Bt cotton. The survival pattern of *S*. *frugiperda* larvae as a function of time was strain dependent (*F*_3,46_ = 3.81; *P* = 0.0199) (Table 1). In fact, the regression curves reveal that the survival response of *S*. *frugiperda* larvae was higher for susceptible larvae than for Cry1F-resistant larvae; however, there was an overlapping of curves at 18 h. At 24 h, the survival rates were 34 and 52% for susceptible and Cry1F-resistant strains, respectively (Fig. 2b). According to the analysis of variance of the proportion of *S*. *frugiperda* larvae found on cotton plants (host acceptance), no significant differences were found between Bt and non-Bt varieties (*F*_*1*,*46*_ = 2.29; *P* = 0.1369) (Table 1). There were no significant interactions among variety, strain and length of time (*F*_*3*,*46*_ = 0.42; *P* = 0.7374) (Table 1). On the other hand, the effect of exposure time on the percentage of larvae found on plants depended on the strain (*F*_*3*,*46*_ = 6.04; *P* = 0.0024). In fact, from 12–18 h, the percentage of larvae of the susceptible strain recovered from the plants significantly decreased compared to that from 0–6 h. In the comparison of percentages of Cry1F-resistant *S*. *frugiperda* neonates recovered from plants over time, there was no difference between exposure times; however, from 12–18 h and 18–24 h, there was a greater percentage of host acceptance than in the susceptible strain (Table 2).

**Table 1:** Summarized model of analysis of deviance for effects of the variety of cotton^1^, strain of insect^2^, and exposure time interval^3^ of neonate to plants on survival of *Spodoptera frugiperda* and analysis of variance (ANOVA) on host acceptance, percentage of neonates that were found on plants and that fed on plant tissues (*IFP*) and percentage of post-feeding dispersal (*PFD*).

Effect	Df	Survival		Host acceptance		*IFP*		*PFD*	
		*F*	*P*	*F*	*P*	*F*	*P*	*F*	*P*
Block	3	0.74	0.5357	0.34	0.7954	12.47	<0.0001	7.72	0.0003
Variety	1	11.90	0.0410	2.29	0.1369	1.40	0.2433	6.64	0.0155
Strain	1	0.26	0.6119	15.15	0.0003	9.10	0.0042	0.49	0.4888
Time	3	1.11	0.2981	0.74	0.5343	3.96	0.0137	0.55	0.6527
Variety *x* Strain	1	1.52	0.2009	1.41	0.2412	7.25	0.0111	1.04	0.3150
Variety *x* Time	3	0.44	0.7254	1.57	0.2100	0.41	0.7467	0.89	0.4533
Strain *x* Time	3	3.81	0.0199	6.04	0.0024	5.08	0.0053	3.11	0.0422
Variety *x* Strain *x* Time	3	0.38	0.7670	0.42	0.7374	0.33	0.8062	0.09	0.9642

^1^Varieties: Bt (WideStrike) and non-Bt near isoline cotton.

^2^Strains: Susceptible and resistant to Cry1F.

^3^Time intervals: 0–6 h, 6–12 h, 12–18 h, and 18–24 h.

**Table 2:** Mean percentage (±SE) of neonates of *S*. *frugiperda* that were found on the plant on Bt cotton and non-Bt cotton at the different tested time intervals. Means within the same time interval column with the same lowercase letter or means between strains within the same row with the same capital letters are not significantly different (*P* = 0.05, Tukey’s test). Original data.

Strain	Exposure time interval of neonate larvae to cotton plants (h)			
	0–6	6–12	12–18	18–24
Susceptible	64.83 ± 4.50Aa	59.00 ± 7.50ABa	33.50 ± 7.70Bb	47.98 ± 9.40ABb
Cry1F-Resistant	62.68 ± 5.30Aa	65.80 ± 5.60Aa	75.80 ± 6.50Aa	73.38 ± 5.50Aa

**Figure 3:**
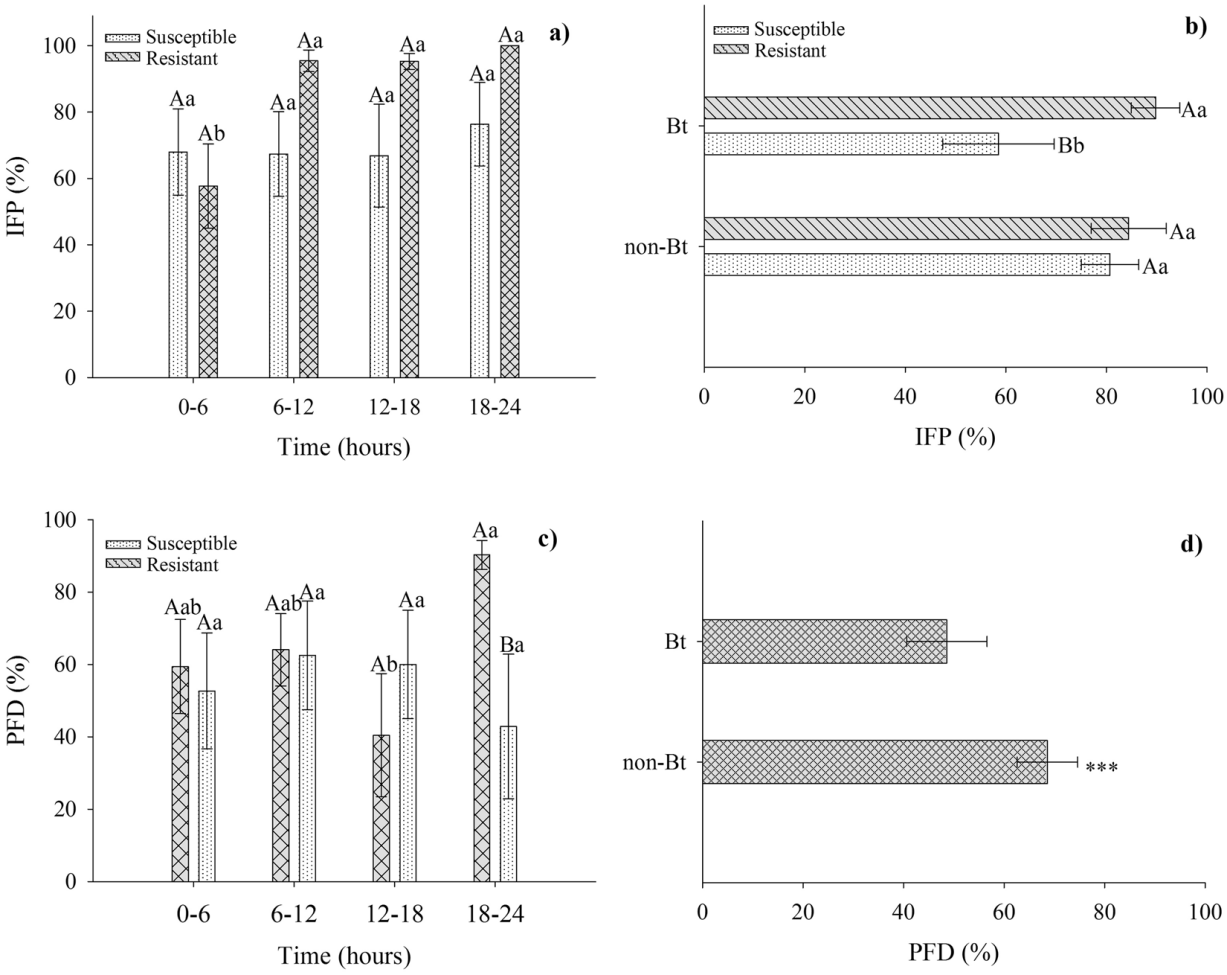
(**a**) Percentage (mean ± SE) of neonates of susceptible and Cry1F-resistant *Spodoptera frugiperda* strains that were found on plants and that fed on plant tissues (*IFP*) at the time intervals 0–6 h, 6–12 h, 12–18 h and 18–24 h or (**b**) that fed on plant tissues from Bt or non-Bt cotton near isoline plants. (**c**) Percentage (mean ± SE) of post-feeding dispersal (*PFD*) of *S*. *frugiperda* strains at different time intervals (**h**) or (**d**) that fed on Bt and non-Bt cotton. Means followed by the same capital letter (comparing bars of the different strains on same axis) and lowercase letter (comparing bars of time (Fig. 3a,c) or variety (Fig. 3c) in the same strain on different axis) are not significantly different as determined by Tukey’s test (*P* = 0.05). ***Asterisks on Fig. 3d represent differences between means. Original data.

The percentages of *IFP* were significantly affected by strain versus time (*F*_*3*,*46*_ = 5.08; *P* = 0.0053) (Table 1); therefore, this variable was summed across Bt and non-Bt varieties. The average percentage of *IFP* for the Cry1F-resistant strain was lower at an exposure time of 6 h than at other exposure times (Fig. 3a). We summed the percentages of *IFP* across all time intervals because of the significant strain-versus-variety interaction (*F*_*3*,*46*_ = 7.25; *P* = 0.0111) (Table 1). The percentage of *IFP* was higher for the Cry1F-resistant strain than for the susceptible strain on Bt cotton (Fig. 3b).

We analysed the effect of the strain-versus-time interaction on the percentage of *PFD* (*F*_*3*,*46*_ = 3.11; *P* = 0.0422) (Table 1), and we observed that exposure time did not affect this percentage for the susceptible strain. However, there was a difference between the Cry1F-resistant and susceptible strains at an exposure time of 18–24 h (Fig. 3c). At exposure times of 12–18 and 18–24 h, we found the lowest and highest percentages of *PFD* for the Cry1F-resistant larvae, respectively (Fig. 3c). The percentages of *PFD* were affected by variety (*F*_*1*,*46*_ = 6.64; *P* = 0.0155) (Table 1); we observed a higher percentage of *PFD* on non-Bt cotton than on Bt cotton (Fig. 3d).

### Feeding behaviour of Cry1F-resistant *S*. *frugiperda* on WideStrike, TwinLink, and non-Bt cotton

**Figure 4:**
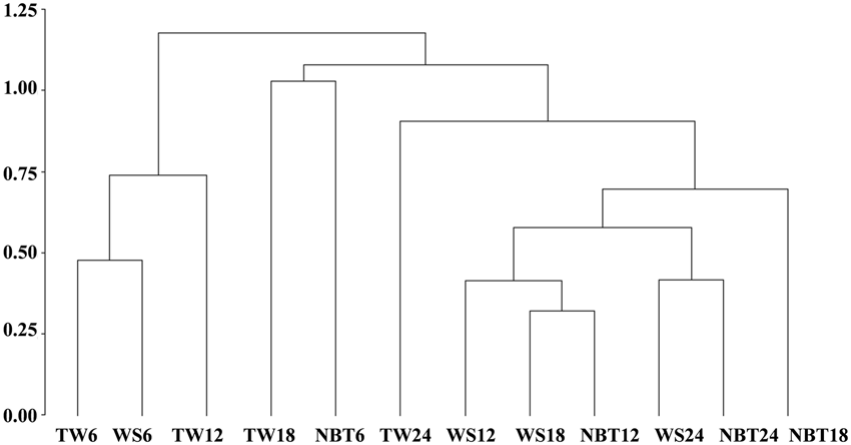
Mean distance between clusters represented by the treatments TwinLink (TW), WideStrike (WS) and non-Bt (NBT) cotton at four time intervals after artificial infestation: 0–6 h (6), 6–12 h (12), 12–18 h (18) and 18–24 h (24). Cluster average linkage method. Distance matrix based on proportion data for survival, dispersal, post-feeding larval dispersal and neonates of *Spodoptera frugiperda* found on plants and that fed on plant tissues.

The distance matrix based on proportion data allowed the observation of treatment clusters by average linkage method. Dissimilarity was studied based on proportion data for survival, dispersal, *PFD*, and *IFP*. This method allowed a comparison between the pattern of feeding behaviour and the susceptibility of *S*. *frugiperda* Cry1F-resistant strains among treatments. By observing the dendrogram from left to right and inserting a cut near 1.15, we observed the existence of two groups inside the cluster that were well-defined based on average distances. This finding indicates that the feeding behaviour and survival of *S*. *frugiperda* are similar when they are maintained on TwinLink (for 6 and 12 h) and WideStrike (for 6 h) (Fig. 4).

By analysing each group from bottom to top, we verified that the feeding behaviour of *S*. *frugiperda* was similar within non-Bt and WideStrike varieties for 12 and 18 h and between these varieties for 24 h in the second subgroup. Following the same procedure led to the observation that the highest dissimilarity occurred with TwinLink for 18 h and with the non-Bt variety for 6 h because there was a longer distance within this group (Fig. 4).

### Movement analysis of the *S*. *frugiperda* strains with video tracking

**Figure 5:**
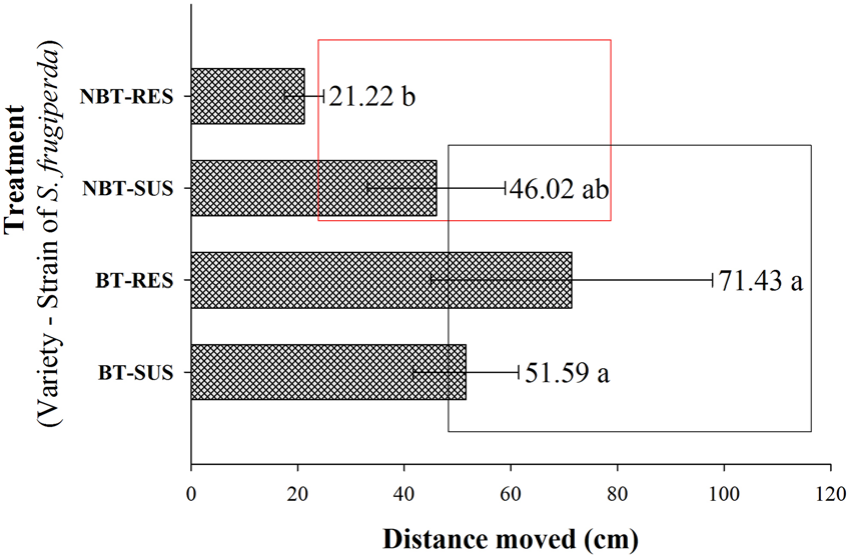
Distance moved (mean ± SE) by susceptible and Cry1F-resistant *Spodoptera frugiperda* strains on Bt and non-Bt cotton. Means followed by the same letters or by rectangles of the same colour were not significantly different as determined by Tukey’s test (*P* = 0.05). Original data.

The mean velocity and continuous mobility period of larvae were not affected by cotton variety (mean velocity: *F*_*1*,*1*_ = 2.61; *P* = 0.2044; continuous mobility period: *F*_*1*,*1*_ = 3.22; *P* = 0.1157) or strain (mean velocity: *F*_*1*,*1*_ = 0.22; *P* = 0.6850; continuous mobility: *F*_*1*,*1*_ = 1.56; *P* = 0.2523). However, the distance moved was affected by cotton variety (*F*_*1*,*1*_ = 8.21; *P* = 0.0242). In fact, neonates of the Cry1F-resistant strain kept on non-Bt cotton moved shorter distances than did larvae of the same strain exposed to Bt cotton (*P* = 0.0129) or in relation to susceptible larvae from Bt cotton (*P* = 0.0452) (Fig. 5). Therefore, the effect of cotton variety on distance moved depends on the strain (*F*_*1*,*1*_ = 4.61; *P* = 0.0500).

### Spatial model

**Figure 6:**
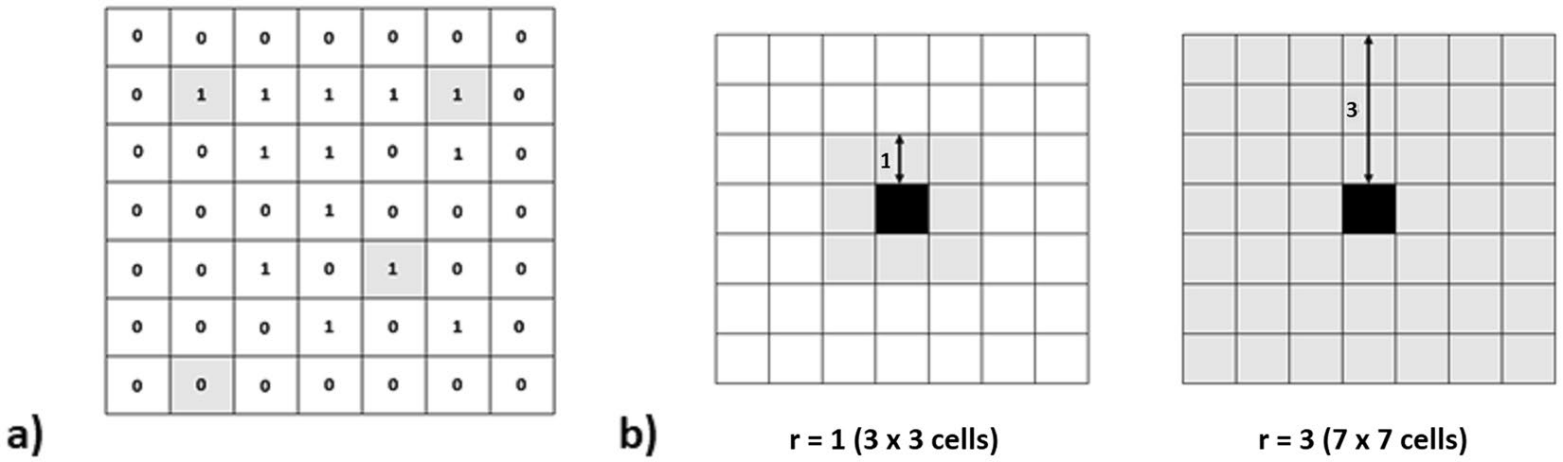
Spatial model representation. Grey cells represent the contamination in a homogeneous landscape. (**a**) Cells receiving a value equal to 0 are empty, and cells receiving a value equal to 1 are occupied by an insect. Cells dynamics are determined by the probability functions. (**b**) Representation of the neighbourhood in which larva dispersal occurred on non-Bt cotton and Bt cotton crops.

**Figure 7:**
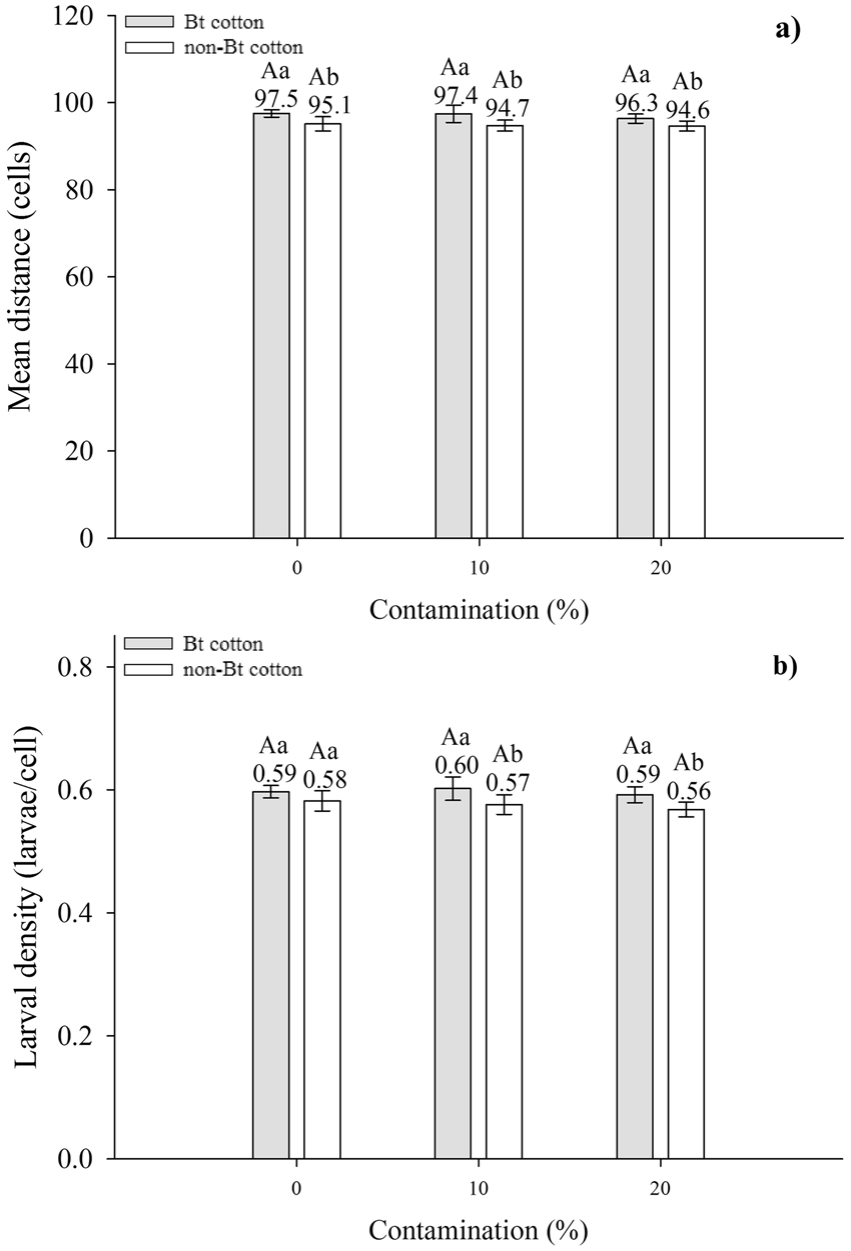
(**a**) Distance (mean ± SE) of *Spodoptera frugiperda* larvae from the centre of the population distribution. (**b**) Larval density (mean ± SE) of *S*. *frugiperda* in the lattice of cells. Means followed by different uppercase letters above contamination levels from the same type of crop and different lowercase letters above different types of crops with the same contamination level were significantly different as determined by Tukey’s test (*P* = 0.05).

Based on data indicating that the movement distance of Cry1F-resistant larvae was approximately three times higher on Bt cotton than that on non-Bt cotton (Fig. 5), we assumed that each Cry1F-resistant larva could move within a region of 7 × 7 cells (radius 3) in Bt cotton varieties and within a region of 3 × 3 cells (radius 1) in non-Bt cotton varieties and represented this movement in the spatial model (Fig. 6). The mean distance of larvae from the centre of the population distribution was significantly different when Bt and non-Bt cotton varieties with the same levels of contamination were compared, but it was similar within the same cotton variety under different levels of contamination (Fig. 7a). Larval density was significantly different only when Bt and non-Bt cotton varieties with contamination levels of 10 and 20% were compared (Fig. 7b).

## Discussion

In the current study, we verified that the dispersal behaviour of the susceptible and Cry1F-resistant strains varied according to the exposure time. The host acceptance rate was higher from 0–6 h after infestation and lower from 12–18 h for the susceptible strain, resulting in high abandonment rates. The Cry1F-resistant strain of *S*. *frugiperda* showed a similar pattern of host acceptance among exposure times. By comparing this trait between strains from 12–18 h and 18–24 h, the highest percentage of host acceptance was found for the Cry1F-resistant strain. Although there is evidence that the dispersal behaviour of Lepidoptera larvae is the result of genetic programming^19,20^, the effect of toxins on survival, combined with larval behaviour, at different exposure times intervals also strongly affects the dispersal rate of neonates^12,19^.

Regarding the preferential response of neonates to feeding on plant tissues, a higher *PFD* was generally found for non-Bt cotton than for Bt cotton. The lowest percentage of *IFP* was recorded on Bt cotton for the susceptible strain. It was common for host plants that contain toxins to stimulate neonate dispersal well before feeding (pre-feeding dispersal) and/or after feeding (*PFD*)^15^, but this was species specific. At an exposure time of 0–6 h, we observed the lowest percentage of *IFP* for Cry1F-resistant larvae. From 0–6 h, there was no difference in host acceptance of *Alabama argillacea* Hübner (Lepidoptera: Noctuidae) larvae between Bt and non-Bt cotton plants^12^. In our study, the highest dispersal rate for the Cry1F-resistant strain associated with plant tissue ingestion was found 18–24 h after plant infestation.

The adaptation of Cry1F-resistant insects to WideStrike and the possible fitness cost found for resistant larvae on non-Bt cotton are likely explanations for the shorter distance moved by *S*. *frugiperda* larvae in the absence of selection pressure (non-Bt cotton) observed via video tracking with EthoVision. According to Vélez *et al*.^14^, there is no strong evidence of differences between susceptible and Cry1F-resistant *S*. *frugiperda* and *Ostrinia nubilalis* (Hübner) (Lepidoptera: Crambidae) strains; however, a low percentage of susceptible strains of both species abandoned the plant tissue expressing Cry1F. Horikoshi *et al*.^17^ showed no associated fitness costs of development time, survival and reproduction variables of the Cry1F-resistant population of *S*. *frugiperda*.

We observed similarity in the feeding behaviour of *S*. *frugiperda* on WideStrike and TwinLink cotton at the beginning of the exposure period (0–6 h) after hatching/infestation. During this exposure time, there was a relatively low percentage of insects that dispersed post-feeding (in comparison to 24 h) and a low number of insects feeding on plant tissues. The high dispersal at the beginning of the exposure time increases the probability of resistance evolution and loss of the TwinLink technology, mainly in agroecosystems where intentional or non-intentional seed mixing occurs (contamination through inadequate agronomic practices such as destruction of cotton plants after harvest, volunteer plant control, and seed saving after harvest). The implementation of strategies based on resistance management aiming to preserve both Bt technologies for WideStrike and TwinLink should use the information generated in the present study concerning the behaviour of the Cry1F-resistant strain of *S*. *frugiperda*. Pyramided cotton containing Cry1Ac and Cry1F was effective against susceptible and heterozygous insects but not against Cry1F-resistant insects^21^. In some cases, chemical control of *S*. *frugiperda* has been necessary in Brazilian agroecosystems using WideStrike because Cry1F-resistant *S*. *frugiperda* survives on WideStrike^22^. TwinLink was effective in promoting mortality of individuals resistant to the proteins Cry1F (in Herculex maize – HX), Cry1A.105 and Cry2Ab (in YieldGard VT PRO - VT), and Vip3 (in Agrisure Viptera - VIP) and of heterozygous individuals from crosses between the susceptible population with strains resistant to HX, VT, and VIP^22^. Technologies combining new insecticide proteins without cross resistance to and high activity against key pests applied in Brazilian agroecosystems or similar systems have been proposed^22,23^. The use of Vip3Aa20 protein has also been recommended for maximizing the durability of Cry proteins in *S*. *frugiperda* control, but tactics based on the high-dose/refuge strategy are necessary to avoid the loss of Vip3Aa20 protein with resistance under field conditions^24^. We would like to emphasize that knowledge of the behavioural ecological traits of *S*. *frugiperda* is also relevant for the establishment of effective resistance management programmes, but it is important to collect data relative to the behaviour of multiple pests^14^, especially in neotropical agroecosystems.

Here, we presented computational support for conclusions concerning the effects of larval movement in different scenarios in Bt and non-Bt cotton fields. Simulations confirmed that the mean distance of larvae to the centre of the population was smaller on non-Bt cotton plants than on Bt cotton plants, irrespective of the contamination degree. Therefore, because of the different rates of larval movement, there was lower expansion of the larval population on non-Bt cotton fields than on Bt cotton fields. However, the levels of contamination did not interfere significantly with the results. These results can be explained as follows: first, in this modelled system, the rate of larval movement was the only variable in the simulations because the model allowed us to isolate the variable of interest. Thus, it should be noted that the model indicated that considering only the differences between the rates of larval movement of *S*. *frugiperda* in Bt and non-Bt cotton would not be sufficient to identify a difference in the dispersion of the whole population at the studied contamination levels (10–20%). Additionally, larval dispersal is a short-scale movement, and therefore, it is expected that its effect on the dispersion of the whole population is only observed when extreme situations are compared (e.g., most of the area contains either Bt or non-Bt crops) and not under slightly different conditions (e.g., different levels of contamination of non-Bt cotton when Bt cotton is the dominant crop).

In conclusion, the present study demonstrated that the dispersal of the Cry1F-resistant strain of *S*. *frugiperda* was affected by cotton varieties over time. The highest rate of *PFD* of Cry1F-resistant larvae occurred after 18 h of exposure; therefore, our results support the idea that the *PFD* at different time intervals is strain specific. In addition, at the beginning of exposure (up to 6 h) of larvae to plants with toxins, there was high degree of similarity in the feeding behaviour between populations of *S*. *frugiperda* kept on WideStrike and those kept on TwinLink. With respect to the sedentary behaviour of the Cry1F-resistant neonates, there is evidence that Cry1F-resistant neonates moved shorter distances when reared on non-Bt cotton than on Bt cotton; in addition, independent of contamination degree, the dispersal capacity of Cry1F-resistant larvae was smaller on non-Bt cotton than on Bt cotton. According to our findings regarding the fitness costs of *S*. *frugiperda* on non-Bt cotton linked to behavioural ecological traits, the present study motivates further research on the expression of genes responsible for the ethological activities of *S*. *frugiperda* strains on different hosts.

## Methods

*Spodoptera frugiperda* and cotton plants were grown at the Insect Ecology and Forestry Entomology Laboratory (ESALQ/USP), Piracicaba, São Paulo, Brazil. We used the susceptible (SS) and Cry1F-resistant (RR) strains of *S*. *frugiperda* characterized by Farias *et al*.^25^. Larvae-rearing stock and plants were kept in a climate-controlled chamber at 25 °C with a relative humidity of 65 ± 10% and a 12-h photophase. Cotton plants expressing the genes for the Bt proteins Cry1Ab/Cry2Ae [variety FM 940 GLT (TwinLink^®^)] and Cry1Ac/Cry1F [variety FM 975 (WideStrike)]^®^ and its non-Bt isoline [variety FM 993] were used in this study. All cotton varieties were planted in plastic pots 25 cm in diameter and 40 cm in height.

### Feeding behaviour of *S*. *frugiperda* strains on WideStrike and non-Bt cotton varieties

This bioassay was conducted to quantify the proportion of *S*. *frugiperda* neonates that dispersed and fed on Bt and non-Bt cotton plants. We used two varieties: the Bt cotton WideStrike and non-Bt cotton (untransformed isoline FM 993). The experiment was conducted in 2 × 2 × 4 factorial randomized blocks with total of 4 blocks, where each block was divided into one combination of strains (SS or RR) and variety (Bt or non-Bt) and four time intervals (6, 12, 18, and 24 h after artificial infestation). We assessed each time interval in different plants independently to avoid repeated sampling in the same experimental unit. The experimental unit consisted of a Bt or non-Bt cotton plant that reached the six-leaf stage and received 20 neonates of *S*. *frugiperda* (0–24 h old) released on a leaf in the apical region of the plant. Each plant was covered with an organza bag. According to each time interval, the bags were inspected, and the larvae were removed with a brush. Each larva was categorized into insects found on the plant or outside the plant. Insects that fed on plant tissues and were found outside the plants were classified as *PFD*, while neonates that fed on plant tissues and were found on plants were classified as *IFP*. To determine whether the larvae had fed, we used microscope slides according to the method adopted by Razze *et al*.^19^ and Ramalho *et al*.^12^.

### Feeding behaviour of Cry1F-resistant *S*. *frugiperda* on WideStrike, TwinLink, and non-Bt cotton

An experimental design similar to that mentioned for the first bioassay was used here. We used one population resistant to Cry1F and three varieties of cotton: Bt cotton varieties (WideStrike and TwinLink) and non-Bt cotton (untransformed cotton variety FM 993). We quantified the following variables: survival, dispersal, *PFD*, and *IFP*; in addition, we calculated the distance matrix based on proportion data.

### Movement analysis of the *S*. *frugiperda* strains with video tracking

We conducted a bioassay to examine the differences between the behavioural traits of *S*. *frugiperda* neonates exposed to Bt and non-Bt cotton plants. We used an experimental design structured as randomized blocks, with two varieties (Bt and non-Bt cotton plants) and two strains (RR and SS) of *S*. *frugiperda*. The movement behaviour of larvae was tracked with automated video-tracking software (EthoVision^®^)^26^ for 12 h.

An infrared camera allowed EthoVision to locate the bodies of *S*. *frugiperda* larvae. Recordings of the paths of individual larvae, so-called “tracks”, were made for each individual larva. We used four replicates (= larvae) per treatment. The experimental unit consisted of 1 larva; each insect was placed in a Petri dish with one leaf of Bt or non-Bt cotton collected at the six-leaf stage. The insects were kept in a climate chamber regulated as mentioned before. EthoVision was used to calculate the variables related to the distance moved by the larvae and to the velocity of neonates. The program generated the following variables: distance moved (cm), mean velocity (cm/s), and continuous mobility period (s)^27^.

### Spatial model

To verify the influence of different movement behaviours studied in first and second bioassays on the distribution of the larval population of *S*. *frugiperda*, we used a spatial model programmed in C and developed by Garcia *et al*.^16^. All probability functions defined by Garcia *et al*.^16^ are presented in Supplementary Equations S1. In this model, a lattice of cells was created to represent the dynamics of immature (larvae and pupae) and adult (only females) insects. Each cell represented either a Bt or a non-Bt cotton plant, and immature and adult insects could occupy the same cell without interfering with each other. In relation to the dynamics of the immature stage, each cell could be occupied by an immature insect (receiving a value equal to 1) or remain empty (receiving a value equal to 0). In relation to the dynamics of the adult stage, each cell could be empty (0) or occupied by a maximum of 10 female adult insects.

A cell occupied by an immature insect in the lattice could become empty due to larval mortality or metamorphosis. Likewise, a cell not occupied by an immature insect could become occupied due to the oviposition of a female adult. A cell not occupied by a female adult could become occupied due to the metamorphosis of an immature insect in the cell. A cell occupied by an adult could become empty due to mortality. We assumed that each adult could randomly move to any cell within a radius of 35 cells from its own cell. In each step, a cell within this radius was sorted out, and in cases where the carrying capacity was not reached, the adult moved towards it.

In this work, six different conditions were simulated by using this model. Within group 1, we had the following conditions: **a1**. resistant individuals in Bt areas without contamination of non-Bt cotton, **a2**. resistant individuals in Bt areas contaminated with 10% non-Bt cotton, and **a3**. resistant individuals in Bt areas contaminated with 20% non-Bt cotton. Within group 2, we had the following conditions: **b1**. resistant individuals in non-Bt areas without contamination of Bt cotton, **b2**. resistant individuals in non-Bt areas contaminated with 10% Bt cotton, and **b3**. resistant individuals in non-Bt areas contaminated with 20% Bt cotton.

The functions were the same in non-Bt cotton and Bt cotton areas. However, larval dispersal was different in each area, considering the results obtained in the bioassay of movement analysis with video tracking. Each simulation was repeated 50 times, and for each time, we calculated the mean distance of larvae represented in the lattice from the centre of the distribution of the larval population after 300 time steps. Larval density was also calculated for each condition.

### Statistical analysis

The data proportions of *PFD*, *IFP*, survival, and dispersal (from the first bioassay: feeding behaviour of *S*. *frugiperda* strains on WideStrike® and non-Bt cotton varieties) were submitted to principal component analysis (PCA). PCA and Pearson’s correlation analysis were performed by following the SAS Procedures^28^ PRINCOMP and CORR, respectively. Regarding the PCA, the data were standardized by dividing the difference of each data point and the arithmetic mean of each variable by the standard deviation of the variable.

Survival data of *S*. *frugiperda* were subjected to analysis of deviance to assess the significance of the interactions among the factors strain, variety and time interval (*P* = 0.05) with a quasi-binomial generalized linear model. The goodness of fit was evaluated using half-normal plots with a simulated envelope^29^ using R^30^. Survival data were also submitted to logistic regression with PROC GENMOD^28^. Additionally, data of *PFD*, *IFP*, and dispersal from the same bioassay were subjected to analysis of variance with PROC GLM^28^ to determine whether there were interactions between factors, and these data were processed through the Box-Cox method^31^. The hypothesis of equality was tested by Tukey’s test (*P* = 0.05).

According to data from the bioassay of the feeding behaviour of *S*. *frugiperda* resistant to Cry1F on WideStrike, TwinLink, and non-Bt cotton varieties, the similarity and clustering treatments were analysed by cluster analysis using the average linkage method and TREE procedures^28^.

The variables calculated by the movement analysis of the *S*. *frugiperda* strains with video tracking were subjected to analysis of variance with PROC GLM^28^ to determine whether there were interactions between strain and variety. The hypothesis of equality was tested by Tukey´s test (*P* = 0.05). The equality between the results of each simulation from the spatial model was tested by using the Tukey-Kramer test (*P* = 0.05).

## Electronic supplementary material

Supplementary Equations
