# Supplementary material for: Larval Dispersal of Spodoptera frugiperda Strains on Bt Cotton: A Model for Understanding Resistance Evolution and Consequences for its Management
Source: Sci Rep. 2017 Nov 23;7:16109. doi: 10.1038/s41598-017-16094-x (PMC5700918; doi:10.1038/s41598-017-16094-x)
Supplement: Supplementary file 1 — Supplementary Equations [file 41598_2017_16094_MOESM1_ESM.pdf]

Larval Dispersal of *Spodoptera frugiperda* Strains on Bt Cotton: A Model for Understanding Resistance Evolution and Consequences for its Management

José B. Malaquias, Wesley A. C. Godoy, Adriano G. Garcia, Francisco de S. Ramalho, Celso Omoto

Supplementary material – Model equations (reproduced from Garcia et al[16])

Survival of resistant immature insects in either non-Bt or Bt-crops (considering a complete resistance and the fitness cost associated only to larval movement)

$$\begin{cases} \mu(i) = 0.98 \text{ if } 0 < i \leq 15 \text{ (corresponding to the larval stage)} \\ \mu(i) = 0.96 \text{ if } 15 < i \leq 25 \text{ (corresponding to the pupal stage).} \end{cases}$$

Metamorphosis (pupa becoming adult)

$$\begin{cases} \sigma(i) = 0 \text{ if } i < 25 \\ \sigma(i) = 1 \text{ if } i = 25 \text{ (at this age, all pupae developed into adults).} \end{cases}$$

Mortality of adult insects

$$\begin{cases} \gamma(a) = 0 \text{ if } a \leq 11 \\ \gamma(a) = 0.1\,(a - 11) + 0.5 \ \text{ if } 11 < a \leq 21 \\ \gamma(a) = 1 \text{ if } a > 21. \end{cases}$$

Oviposition by a female adult

$$\begin{cases} \phi(a) = 0 \text{ if } a \leq 3 \\ \phi(a) = 0.7\left(\left(\frac{1}{e^{0.3\,(a-1)}-0.3}\right) - \left(\frac{1}{e^{0.3\,a}-0.3}\right)\right) \text{ if } a > 3. \end{cases}$$
